# Supplementary figures and images for: Screening, Characterization and Comparison of Endoglucanases/Xylanases from Thermophilic Fungi: A Thielavia terrestris Xylanase with High Activity-Stability Properties
Source: Int J Mol Sci. 2025 Jul 17;26(14):6849. doi: 10.3390/ijms26146849 (PMC12295501; doi:10.3390/ijms26146849)

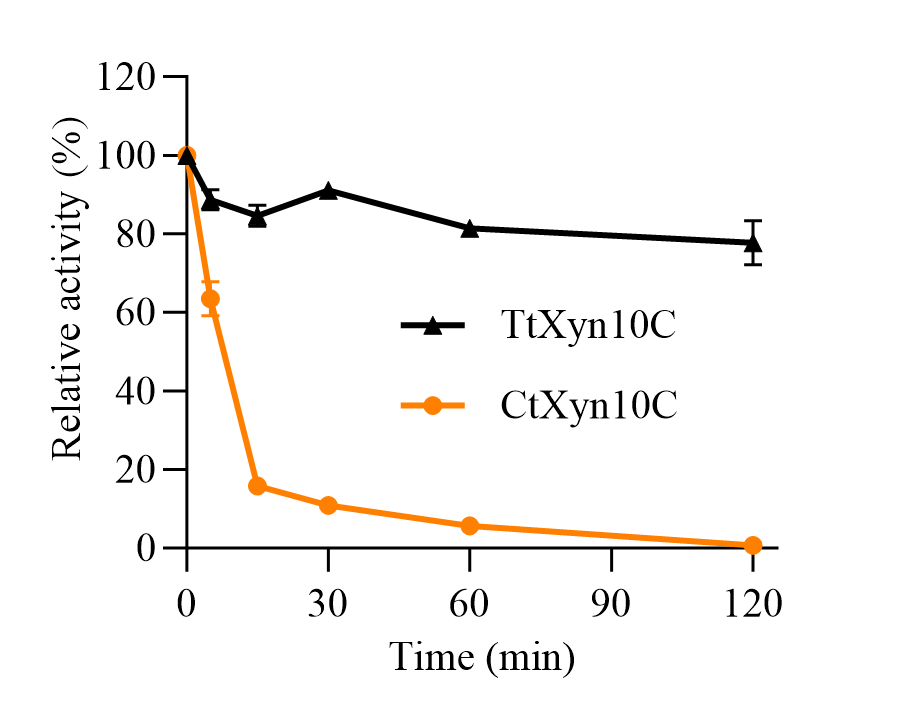

Supplement: Supplementary file 1 [file ijms-26-06849-s001.zip › ijms-3740238-supplementary.tif]
